# The Preservation of the Teeth Treated as a Specialty

**Published:** 1876-02

**Authors:** E. D. Miles

**Affiliations:** Philadelphia, Pa.


### THE PRESERVATION OF THE TEETH TREATED AS A SPECIALTY.

BY E. D. MILES, M. D., PHILADELPHIA, PA.

*Read before the American Academy of Dental Surgery, New York, October 19th, 1875.*

All the attention heretofore given to this department has been simply an *incidental* feature of dentistry. But if it is a field large enough and sufficiently important to occupy the whole attention of any one practitioner, under the *aegis* of due qualifications including especially long experience and success, why should it not be *occupied as such?*

Division of labor, in most of the avocations, has been found favorable to high special attainments. Experts are usually found devoting to a department their whole time and most assiduous attention. Even the barber has employes who are considered and likely to be petted, as *adepts*, in one particular feature of his business. A good hair cutter is not expected to give so clean a shave, so smooth and unruffling to the nerves, as one who wields only the sharp and well poised razor, but is always expected to produce a fine shaped head and "get up" a most delightful champoo.

A very fine plugger or operater ought never to touch a plate, it spoils his hands for the acute sense of *touch*, needed in the delicate manipulations, while operating upon sensitive teeth and cavities, to say nothing of delicate faces and fastidious tastes, among many of his patients.

A hobby in any art or avocation is the most constant incentive to improvement. A desire to excel in any particular direction, generally results in the most laudable as well as profitable achievements; which all men of culture and systematic thought, may well encourage till the highest pinnacle of perfection shall have been attained and he who gives his whole attention, with what natural gifts he may bring to bear upon the subject, will be the most sure to reach it. The operator, observing anything less than the best condition of the patient's mouth, says: "Why don't you take better care of your teeth? You don't brush them enough,—get a good stiff brush and brush away till your teeth are clean, and keep them so, and you will find that your teeth will not decay so rapidly nor decay away from my fillings especially, which, no matter how well put in, have no guarantee for permanence, as long as they are left to the chance of contiguous decay." Often the patient will answer: "I do brush my teeth doctor and too with a very stiff brush, till they sometimes ache, and still they are not clean and nice, as I'd like to have them, I have brushed them after every meal, all seemingly to no good and no effect; except to brush the gums away from my teeth, leaving the necks bare and tender." "Well you use such and such powder and brush this way and that, crosswise, up and down," etc., etc. They separate: One, the dentist, with an honorable sense of having acquitted himself of a duty, too often observed in the breach, but frequently bespoken, by any quantity of similar and slightly differing cases. The patient goes home with a renewed determination to show better teeth the next time they return for the inspection or work of the dentist, still they fail in exhibiting that perfect condition, as to purity, health and real beauty, inherent in nearly all teeth, but which their imperfect knowledge and efforts failed to bring out. Now I am not going to say here that the dentist does not give the proper instruction, nor sufficient instruction or in the right direction, but I do say, that if the same dentist, or artist, as I feel disposed to consider many of them, and of a very superior order, should devote his entire attention, with the same preparatory education and professional knowledge, to this one department with corresponding zeal and pride in his work, he would be far more successful and efficient. But not one of these patients having paid well for this special consideration, he sees no adequate inducement to give all the necessary time and study to the preliminary treatment required for a fair commencement or ground work, as well as for the subsequent daily care, the constant and special supervision; working with the aid of ingenious materials, collected for use for one period and intermitted for another, alternated, repeated or exchanged, as the different and progressive phases of amelioration and improvement are brought forward, till it will finally dawn upon the mind of the intelligent and appreciative patient, that they are not only following, but learning the true rationale of a system, well digested and tried; for the highest development, perfection and care of the teeth, showing a new beauty and increased health, never enjoyed before.

This argument of special attention is well understood when applied to collateral arts, and may just as reasonably be brought into requisition—for the no less important subject,—but far more neglected system, for the preservation of the teeth.

The protection and preservation of good teeth, together with the restoration of all conditions of imperfect teeth; from incipient decay to the largest havoc in that direction, should be the legitimate field of the specialist and his attention and supervision should be among the earliest and latest, in fact always in demand.

The idea of confining him to the consideration of long neglected and exaggerated disease, where for instance, the alveolar processes have been seriously invaded, if not entirely destroyed, with the expectation that he shall restore them or relinquish all claims to skill, for the conservation of the teeth; in very many different abnormal conditions, needing skillful treatment and care, long before reaching that fatal point, when a process or periosteal membrane must be restored to a bone, as smooth as glass, with every vestige of attachment and growth completely obliterated. Such rule or exaction, is not only unfair but preposterous. It would stultify every reasonable claim for a practical benefaction and deprive all alike from the least possible chance to practically illustrate any merit whatever.

The most apparent efforts, for the preservation of the teeth, have been pretty generally monopolized by enterprising venders of empirical compounds and tooth lotions; made up and christened with very hard and enigmatical names and sent broadcast; whose persistent advertisements are the common ornament, of all the otherwise unoccupied fences and rocks in the country. If the stuff was cheap, it was the chief recommendation to the many (who don't neglect every thing,) If put up prettily in gay looking packages, it quite as well answered the more exacting *beau monde* to say nothing of the *demi monde*, who have been known to set the fashions in some countries and give celebrity and *eclat*, to articles equally meretricious as themselves, while the more cautious and intelligent endeavor to rely upon the resources of the profession, where they not unfrequently, have also failed—measurably at least, or to the extent that it was not so thoroughly successful as that secured by the constant if not ingenious supervision of the better prepared, and more experienced specialist; whose manipulations, treatment and care, have not been made less efficient with collateral claims upon his time and ingenuity.

Too many employments must necessarily lesson and divide the efficiency of the artist, artisan or professional exemplar of science, especially will it distract the attention and discernment of things, pertaining to the peculiar idiosyncrasies and daily habits, and changing caprice, which failing humanity so often exhibit; where they are compelled to rely upon some outside advice and direction, and just as often, provokingly neutralized, by idle neglect or whimsical indifference. Nothing less than the most direct and pointed rules will suffice, reiterated and constantly supervised and revised, to see to their faithful observance, mitigated in their apparent hardness and inflexibility with kindly and easily comprehended explanations, of the rational philosophy and utility of the course; so as to fully sustain the resolution of the sometimes doubtful patient to persevere till they can as fully comprehend practically and theoretically its beneficence, truthfulness and beauty, thus realizing all promised by the artist or desired by the patient.

Absolute purity should be the basis upon which all subsequent treatment must be founded. To establish this necessary foundation, the cleansing process should be effected by means differing as much as the different phases of discoloration, accumulation of tartar and rust, to use a common phrase, upon the teeth, as well as the different conditions of the gums from simple irritation, spongy and softening to ragged, congested, phagedenic ulcers, which are frequently denominated in common parlance, oral scurvy; often exhibiting saneous discharges by simply rubbing the finger across the gums. Of course these require various topical treatment, some of powerful action, as the dilute reagents, escharotics and stiptics down to antiseptic astringents, all acting as stimulant tonics and alteratives, to the more gentle and appeasing anodynes and fragrant stimulants, all of which can be accomplished, if done properly, without pain. In the subsequent treatment and for the use of the patient, different materials of erasive and detersive qualities; sometimes cutting but most discriminatingly used, down to an impalpable polish and neutralizer, to answer the different conditions and constitutional habits, whether of an acid or alkaline reaction, all cleansing, polishing, hardening and strengthening the parts, the bony as well as the soft tissues. The teeth to be pure, hard and beautiful; the gums dense and strong enough to hold them well and firmly in their sockets. No more impurity, no more bad breath; a great indemnity and relief when previously endured. Preparations of whatever quality for the teeth, can not be sold over a counter and used to the greatest benefit, nor even with safety; for in cases where they have been supposed to have been effectual, there is no guarantee whatever, that they will remain so or that they should not be superceded by another and applied by the specialist; who not only watches the progress of its effects, but is responsible for any misapplication and whose watchfulness will enable him to know the constant workings of all the particulars of the treatment and care, as they progress from month to month and from year to year as long as they live.

A simple brush alone has done as much harm, for want of knowing its true use as the neglect of it. Especially a stiff one, and that too, without a particle of intervening material, which is far better in its use than in its neglect; if of the right kind, at the right time and in the right manner.

And that is the system which the specialist, if born under the right star, with the right inclination, genius and training, as well as experience, will certainly discover, and bring into practical operation, according to his chances and opportunities to be a benefactor, where the talent is usually scarce among his fellow beings. Every man and every woman's experience will, I believe, bear me out in the assertion.

There is no one brush that will suffice under any treatment. There should always be two brushes, for the mouth and teeth. An outside and inside brush. The former need not take any of the very odd shapes, so rife to do its best purpose. It should be plain and elastic, never over stiff and hard. I have seen thousands made and called their best brushes, entirely unfit for any conservative use whatever; they tear and lacerate the gums or produce excessive absorption. It is no small thing to know what kind of a brush and then how to use it without injury, and only for the greatest good. It can not be told nor written, and can only be taught the patient from day to day. The more apt to learn, the sooner will the knowledge be acquired. The teacher being also apt in imparting instruction, both can "flourish like a green bay tree" although some prefer to apply it to "other green things" and that might well apply some people's teaching, if not to some who are in the attitude or ought to be of learner. "How not to do a thing," however, is a pretty fashionable ingredient, now a days, notwithstanding Dickens' graphic pencilings of the Circumlocution Office. Besides what that genius discovered and wrote, we have found another component, not inaptly styled, certain kind of unmentionable obstinacy, that would seem to conserve no interest, but those whose chief aim was always how to do it.

The inside brush, perhaps, as such matters stand, might be reckoned the more important of the two—as we think best to teach our patients; because naturally the most likely to be neglected. It is only about one and a quarter or one and a half inches long, and never less than five rows; wire drawn and waxed in, flat curved handle, the brush on the inside of the curve. It will reach all the parts of the inside or lingual portions of the teeth. It should be made of good stiff bristles very unlike the outside brush, which is used on the outside or buccal surface of the teeth, which being convex, must be approached with far less force or leverage to avoid too much friction and the ridging of the external surface of the teeth, as well as wearing the necks into grooves or gutters and driving the exposed gum away from the same and giving them an exaggerated length, sometimes causing a sensitiveness in the denuded necks, that is exquisitely painful to the touch; often calling forth an expression to indicate the exposure of a nerve. Of course it is only an encroachment upon the sensitive dentine, which sometimes rivals an exposed pulp.

The inside brush, coming in contact with the inside or concave surface, will bear a leverage of ten times greater force than the outer or concave surface, as it will also, of a far more cutting and erosive material, never to be used except when positively needed however, for cleansing the mouth and removing the daily accumulations of tartar, which must never be allowed to remain, as it sometimes does till it forms in spite of a certain amount of inefficient brushing, a hard adamantine crust, that resists any and all brushing, and which nothing less than the scaler, sometimes softened and aided by a judicious use of a dilute reagent, which must always be exhibited with great caution and followed by compensating neutralizers and thorough washing to avoid the calcareous affinities and asperity of surface liable to follow, but always desirable to remove; leaving the tooth perfectly intact and smooth as a well polished gem. A stick should be used and considered one of the most s ential aids, in removiug either food or the salivary deposits from the teeth where no brush can reach it, and where efforts to accomplish it result only in overdoing the whole thing, as well as the up and down movement of the brush, quite as liable to imperil the integrity of the edges of the gum; for what is down to one is up to the other, and but little security in using the brush in that manner, and indeed no necessity. The primary art to attain in caring for the teeth, is to secure as much indemnity as possible, with the least possible application of means; for every effort is aggressive and a certain amount of the integrity of both teeth and gums will suffer, just in proportion as you go beyond the *juste milien* or golden mean, in the use of all tried materials or falling short of it.

The subject is, therefore, susceptible of improvement by great care and study; being always bound to take no step without a reason to yourself and faithfully imparted to your patient, who is in reality, the most active and interested partner in the compact; as it most undoubtedly is a compact between teacher and pupil, the terms being, that as long as the teacher shows no remissness in the teaching, the pupil shall manifest the most lively obedience and good faith, in all the particulars, involving reciprocal duties. Under such circumstances there will be hope for pure, healthy and beautiful teeth during life, otherwise, ever changing phase, and go down to old age, till they meet together in that narrow house which is but the threshold of that sphere, where there are no limits to the freedom of a faithful spirit; whose study of the pure and beautiful here will find its counter part beyond, where the still more beautiful is realized and where the true desire shall be perfectly understood and the progress of the soul unimpeded, by error and misconception, shall always rise higher and higher towards the great vertex of the beautiful—the God of the universe.

The improvement wrought in the condition of the teeth, when under its most appropriate and assiduous treatment and care, as a specialty, is, according to our experience in many years practice and observation, very apparent, far excelling in beauty and health, anything I have yet seen, secured from the incidental and partial attention of the general dentist, and still more under the independent care and attention, as picked up and applied, from the various hints and practice found among the resources of the household. The increased beauty alone without reckoning the unfailing health and comfort, is quite sufficient inducement for all lovers of the pure and beautiful to undertake the necessary preliminary treatment; and cheerfully devote five minutes a day in the right direction in the care of their teeth. The time not being the principal ingrediant, but the way, the mode and the means, and instrumentalities used for maintaining a perfection that not only proclaims this wonderful beauty which may be retained for life, but fosters health and comfort quite as uncommon, and superior, as the more apparent and extraordinary beauty. Really, none but the most idle and heedless would choose any other means unless beyond their power to secure its benefit. If it would not be impertinent to still refer to personal experience, we are free to say, that we have seldom seen any teeth, if at all, ranging through all the grades of best to worst, which our treatment would not improve in appearance, and which our system of care, which can only be learned by practice, each for himself, perfected as a specialty, would not maintain if strictly followed and observed.

We have never seen gems, which we naturally admire for their own inherent beauty, when artistically developed by the lapidary, more gratifying to look upon than very many teeth once supposed to be so poor as to be beyond the art of any treatment, recalled to a health and a pristine beauty, that the proudest might envy, as a source of increasing delight and admiration. I hope the writer of this paper will be again excused if he adds that he would esteem it a great privilege if opportunity should be afforded him by the profession, while visiting New York, as he may periodically, to demonstrate practically, how much and how beautiful an improvement can be made in this direction.

Tehe oft repeated remark that "dentists are not interested in the preservation of the teeth," would be as invidious as a similar one, that "doctors rejoice when the people are sick," and need not be quoted here except in this connection, to add my testimoney as far as it goes, to correct the mistake; for there is undoubtedly, no step that the dentist takes, in his profession, that is free of the hope and intention to save the teeth, except when he extracts them; and then often, at the impatient and ill-starred importunity, if not imperative demand of the patient; defying all the consequences, so that it relieves them from a present unbearable pain; no matter how much mortification and regret it may bring them in the end. As I understand, it is the fashion, to say nothing of the duty, to try to save the teeth, and some most marvelous work of the operator has been done for that purpose when nothing but imperfect sections of the crown have been left on which to build up a complete, systematical artificial gold crown, one of the most signal triumphs of the dental artist. Still a well preserved hard and brilliant natural tooth, crown and all, must and will undoubtedly be always preferred.

It might be well to advert to another fashion, that contemplates the loss of teeth, merely because it can be effected without pain, a wondrous specimen of economy, which might as well induce people to have their teeth extracted, as has often occurred, for fear that they may ache at some future day! We can not avoid the reflection, that at some distant age—say one thousand years hence, it is just possible that these days of anæsthetic exemption from pain, will be recalled as the second age of classic heroes; not precisely like the old days of *Spartan glory*; when her boys were taken to her sacred altars and there scourged with whips till the blood ran down their heels, not a murmur escaping their lips, to accustom them to bear pain with heroic fortitude. And it was such boys, grown up to manhood, under public discipline and self-abnegation, who combed their hair in sight of the enemy before the battle, whose patriotic heroism served them as well, when, at the pass of Thermopylae, 300 strong, they opposed the invading hosts of the haughty Xerxes till every one was slain but the last man, who then fled, (leaving 20,000 dead Persians upon the field,) to carry the news to Greece, of the heroic sacrifice and self-immolation of his fellows upon the altar of his country; but whose disgrace for leaving it alive was not removed till he regained it in another noted battle. We wonder what chance "nitrous oxide" would have had in those days when to bear pain was an honor, sought for and won with a courage; presenting a strange contrast with the present times, when people even give up "their very teeth," for fear that on some unlucky day they may chance to ache? We are suspicious that if that renowned Spartan band of heroes could be recalled to life and to the field of modern anæsthesia, witnessing the sacrifices here performed, of an odontological character, they would fain exclaim, as of old, when addressing the runaway messenger from the field of that celebrated Phosian Pass, "*See the trembler—Austodemus.*" Verily, such fearful people, where pain so thoroughly demoralizes them, should learn at their earliest opportunity, the very best way to preserve their teeth—sound and beautiful—then will they escape both penalties, the excruciating pains and irreparable losses.

An immunity from the ordinary chances of decay of the teeth, should entitle the artist to a consideration not easily cancelled. Money has a very soothing effect upon exacting merit, and in the parlance of the times, "the more the better," we believe to be the natural response of all who know the wondrous power and uses to which that article is put. Still we feel, for ourselves, that if the obligation ceases with the signing of a receipt, that we do not prefer that mammon should be our presiding deity and we should seriously think of compromising an over estimate in gold, be diluting it with "just a spice of" gratitude. He who settles such debts with money alone, "pays trash" still worse, the empty thanks of "dead hearts,"(!) but if he pays well, of his own motion, he gives significance of his gratitude and a true sense of appreciation within. To refuse all offered consideration, even if in the medium, borne upward and downward, on the hallowed precincts of the gold room, (!) might give rise to the suspicion, that all was right but the head!

